# Human Astrovirus 1–8 Seroprevalence Evaluation in a United States Adult Population

**DOI:** 10.3390/v13060979

**Published:** 2021-05-25

**Authors:** Lena Meyer, Kevin Delgado-Cunningham, Nicholas Lorig-Roach, Jordan Ford, Rebecca M. DuBois

**Affiliations:** Department of Biomolecular Engineering, University of California Santa Cruz, Santa Cruz, CA 95064, USA; lenameyer@ucsc.edu (L.M.); kemdelga@ucsc.edu (K.D.-C.); nlorigro@ucsc.edu (N.L.-R.); jordanc4rd@gmail.com (J.F.)

**Keywords:** human astrovirus, seroprevalence, biolayer interferometry immunosorbent assay

## Abstract

Human astroviruses are an important cause of viral gastroenteritis globally, yet few studies have investigated the serostatus of adults to establish rates of previous infection. Here, we applied biolayer interferometry immunosorbent assay (BLI-ISA), a recently developed serosurveillance technique, to measure the presence of blood plasma IgG antibodies directed towards the human astrovirus capsid spikes from serotypes 1–8 in a cross-sectional sample of a United States adult population. The seroprevalence rates of IgG antibodies were 73% for human astrovirus serotype 1, 62% for serotype 3, 52% for serotype 4, 29% for serotype 5, 27% for serotype 8, 22% for serotype 2, 8% for serotype 6, and 8% for serotype 7. Notably, seroprevalence rates for capsid spike antigens correlate with neutralizing antibody rates determined previously. This work is the first seroprevalence study evaluating all eight classical human astrovirus serotypes.

## 1. Introduction

Astroviruses are a diverse family of small, nonenveloped, positive-sense RNA viruses that infect mammalian and avian species [1]. Astrovirus infection is linked to a variety of disease manifestations, growth defects, and mortality in poultry [2]. In mammals, astrovirus infection mainly causes viral gastroenteritis but can also be asymptomatic [3,4] or cause neurological syndromes and encephalitis in rare cases [5,6,7,8]. Human astroviruses are classified into three clades: classical serotypes 1–8, where serotype 1 is the most prevalent globally [9,10,11], as well as the emerging serotypes MLB and VA [5,8].

Human astroviruses (HAstVs) are a leading worldwide cause of viral gastroenteritis but are among the most poorly characterized enteric viruses [12]. Young children, the elderly, and the immunocompromised are most threatened by astrovirus infection, especially in developing countries [13,14,15,16,17,18,19,20]. Worldwide, human astrovirus infection accounts for approximately 2 to 9% of all acute non-bacterial gastroenteritis in healthy children [21]. The United States sees an estimated 3.9 million cases of astrovirus gastroenteritis each year [22]. However, immunofluorescence studies have demonstrated that about 75% of healthy adults have anti-astrovirus antibodies targeting at least one of the eight classical serotypes [23], and another investigation showed that seroprevalence of neutralizing antibodies increases with age [10]. These findings suggest that cases of astrovirus gastroenteritis may be undercounted and that astrovirus disease may actually be a common childhood infection.

Several lines of evidence highlight the importance of anti-astrovirus antibodies developed in childhood in preventing reinfection in adulthood. Firstly, astrovirus infection is rare in adults [23]. Although approximately 75% of adults have anti-astrovirus antibodies, clinical investigations in healthy volunteers determined that more severe disease is associated with seronegativity for anti-astrovirus antibodies [24,25]. Finally, immunoglobulin replacement therapy resolved a persistent human astrovirus infection in an immunocompromised patient [26]. These studies indicate that a vaccine and therapeutic antibodies could be developed to prevent and/or treat human astrovirus gastroenteritis.

**Figure 1:**
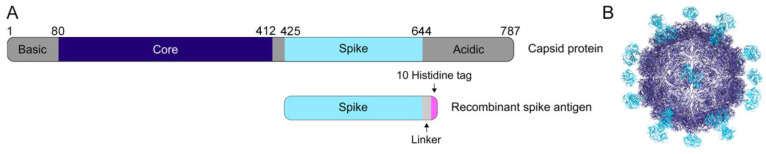
The human astrovirus capsid. (**A**) Schematic of the human astrovirus capsid protein and the recombinant spike antigen used in this study. (**B**) The mature human astrovirus particle, with the spike in cyan and the core in dark blue (adapted from [28]).

Previous structural studies have defined the structural domains of the human astrovirus capsid (Figure 1A,B) [27,28,29,30]. The capsid core domain, which forms the structural icosahedral shell encapsulating the viral genome, remains highly conserved across all eight classical serotypes, ranging from 83.3–97.0% sequence identity between any two serotypes (Table 1). In contrast, the capsid spike domain, which forms dimeric protrusions on the surface of the viral particle, is quite variable, with a spread of 41.4–75.7% sequence identity between any two serotypes (Table 2). Prior work using enzyme-linked immunosorbent assays showed that both the core and spike domains are antigenic [28].

Serosurveillance investigations bring the frequency of previous human astrovirus infection in a particular cohort into focus, but only a few have been performed on specific astrovirus subsets (classical serotypes 1–7, MLB1, HMOAstV-C (VA1), or turkey astrovirus 2) or in specific populations (turkey growers and abattoir workers) (Table 3) [10,23,31,32,33,34,35,36]. Many of the studies focusing on classical human astrovirus were conducted more than 20 years ago. Importantly, no previous serological survey has evaluated seropositivity to all eight classical human astrovirus serotypes. In addition, these studies used a variety of human astrovirus antigens to determine seroprevalence, including mature human astrovirus particles, the full capsid protein, or capsid protein fragments (Table 3). However, studies that evaluate reactivity to the whole capsid are limited because of the possibility of cross-reactive antibodies that may recognize the conserved core domains of several serotypes. The use of the more variable capsid spike domain as the antigen provides an opportunity to confidently evaluate serotype reactivity across all eight serotypes. Here, we provide an updated report on the seroprevalence of each of the eight classical serotypes in a United States adult population, as determined by biolayer interferometry immunosorbent assay (BLI-ISA) using recombinant human astrovirus 1–8 capsid spikes (Spike 1–Spike 8) as the antigens [37].

## 2. Materials and Methods

### 2.1. Human Astrovirus Capsid Sequences Used for Sequence Identity Studies

The accession numbers of sequences used for sequence alignments of the human astrovirus capsid core are as follows for serotypes 1 to 8 (Core- accession number: residues (depository)): Core 1- AAC34717.1: 80-410 (Genbank), Core 2- AZB52195.1: 80-409 (Genbank), Core 3- Q9WFZ0: 82-412 (Uniprot), Core 4- Q3ZN05: 80-410 (Uniprot), Core 5- Q4TWH7: 79-409 (Uniprot), Core 6- AZB52207.1: 80-410 (Genbank), Core 7- Q96818: 81-411 (Uniprot), and Core 8- Q9IFX1: 80-410 (Uniprot). The accession numbers of sequences used for sequence alignments of the human astrovirus capsid spike are as follows for serotypes 1 to 8 (Spike-accession number: residues (depository)): Spike 1-AAC34717.1: 430-644 (Genbank), Spike 2-KY964327.1: 12-228 (Genbank), Spike 3-Q9WFZ0: 432-645 (Uniprot), Spike 4-Q3ZN05: 430-644 (Uniprot), Spike 5-Q4TWH7: 429-641 (Uniprot), Spike 6-AZB52207.1: 430-642 (Genbank), Spike 7-Q96818: 431-644 (Uniprot), and Spike 8- Q9IFX1: 431-644 (Uniprot).

### 2.2. Reagents and Supplies

Anti-Penta-His (HIS1K) sensor tips (Sartorius ForteBio, Goettingen, Germany, 18-5120), Benzonase (Merck Millipore, Burlington, MA, USA, 71205), bovine serum albumin (BSA) (Fisher, Hampton, NH, USA, BP1600), CD OptiCHO expression medium (Gibco, Thermo Fisher Scientific, Waltham, MA, USA, 12681-029), CHO CD EfficientFeed A (Gibco, Thermo Fisher Scientific, Waltham, MA, USA, A10234-01), ChonBlock (Chondrex, Woodinville, WA, USA, 9068), 4 nm Colloidal Gold-AffiniPure Goat Anti-Human IgG Fcγ fragment-specific (Jackson ImmunoResearch, West Grove, PA, USA, 109-185-170) rehydrated in 1 mL deionized water per the manufacturer’s instructions, 4 nm Colloidal Gold-AffiniPure Goat Anti-Rabbit IgG (H + L) (Jackson ImmunoResearch, West Grove, PA, USA, 111-185-144) rehydrated in 1 mL deionized water per the manufacturer’s instructions, glucose (Sigma Aldrich, St. Louis, MO, USA, G8769), 1 mL HiTrap Protein A HP column (GE Healthcare, Chicago, IL, USA, 17-0402-03), HT supplement (Gibco, Thermo Fisher Scientific, Waltham, MA, USA, 11067-030), L-glutamine (Gibco, Thermo Fisher Scientific, Waltham, MA, USA, 35050-061), phosphate buffered saline (PBS) tablets (Sigma Aldrich, St. Louis, MO, USA, P4417), Pierce IgG Elution Buffer (Thermo Fisher Scientific, Waltham, MA, USA, 21004), Pierce Normal Rabbit Serum Control (Thermo Fisher Scientific, Waltham, MA, USA, 31884), Pierce Protein A IgG Binding Buffer (Thermo Fisher Scientific, Waltham, MA, USA, 21001), Pluronic F68 (Gibco, Thermo Fisher Scientific, Waltham, MA, USA, 24040-032), protease inhibitor cocktail set V EDTA-free (MilliporeSigma, Burlington, Massachusetts, USA, 539137), sodium butyrate (Sigma-Aldrich B5887), Superdex 75 10/300 and 16/600 (Cytiva, Marlborough, MA, USA), TALON metal affinity resin (GE Healthcare, Chicago, IL, USA, 28-9574-99), tilted-bottom (TW384) microplates (Sartorius ForteBio, Goettingen, Germany, 18-5080), Tween-20 (Fisher, Hampton, NH, USA, BP337), yeastolate (BD Biosciences, San Jose, CA, USA, 292804).

### 2.3. Expression and Purification of Human Astrovirus Spikes 1–8

Synthetic cDNA encoding Spike 1 (residues 429-645, accession AAC34717.1 (Genbank)), Spike 2 (residues 12-228, accession KY964327.1 (Genbank), a partial HAstV2 capsid sequence), Spike 3 (residues 431-647, accession Q9WFZ0 (Uniprot)), Spike 4 (residues 429-646, accession Q3ZN05 (Uniprot)), Spike 5 (residues 428-644, accession Q4TWH7 (Uniprot)), Spike 6 (residues 429-644, accession AZB52207.1 (Genbank)), Spike 7 (residues 430-646, accession Q96818 (Uniprot)), or Spike 8 (residues 429-647, accession Q9IFX1 (Uniprot)) were cloned separately into pET52b (EMD Millipore) in frame with a C-terminal linker, thrombin cleavage site, and 10-Histidine tag. The plasmids were verified by DNA sequencing. Spike plasmids were transformed into *E. coli* strain BL21 (DE3). Expression was induced with 1 mM IPTG and carried out at 18 °C for 16 h. Cell pellets were resuspended in 20 mM Tris-HCl pH 8.0, 500 mM NaCl, 20 mM imidazole (Buffer A) containing 2 mM MgCl_2_, 0.0125 U/μL benzonase, and 1x protease inhibitor cocktail set V EDTA-free. Cells were lysed using ultrasonication, and spikes were batch purified using TALON metal affinity resin. Elutions were carried out using Buffer A with 500 mM imidazole. Spikes were dialyzed into 10 mM Tris-HCl pH 8.0, 150 mM NaCl (TBS) with 1 mM DTT and purified using size exclusion chromatography in TBS with a Superdex 75 16/600 (Spikes 1, 2, 5, 7, and 8) or Superdex 75 10/300 column (Spikes 3, 4, and 6).

### 2.4. Expression and Purification of the SARS-CoV-2 RBD

The SARS-CoV-2 receptor-binding domain (RBD) in frame with a 10-Histidine tag and AviTag at the C-terminus was expressed and purified, as described in [37].

### 2.5. Expression and Purification of Recombinant mAb 3B4

Mouse hybridoma cells producing mAb 3B4 were generated, as reported in [38]. The amino acid sequences of the mAb 3B4 variable regions were identified as described in [39], allowing for recombinant antibody expression. Synthetic cDNA encoding the 3B4 kappa and heavy chain variable regions (Integrated DNA Technologies, Coralville, IA, USA) was cloned by Gibson assembly into the two pCMV-VRC01 antibody backbone vectors for light and for heavy chains, which contain the constant regions of VRC01, a human anti-HIV antibody targeting the gp120 protein [40]. Cloned sequences were in frame with an N-terminal secretion signal sequence. The resulting expression plasmids, pCMV-VRC01_3B4_kappa and pCMV-VRC01_3B4_heavy, contain the variable regions from the original mouse antibody 3B4 and the constant regions from a human IgG1 antibody under the control of the human cytomegalovirus promoter. The plasmids were verified by DNA sequencing. The expression plasmids were used in a 1:1 ratio to electroporate Chinese Hamster Ovary suspension (CHO-S) cells using the MaxCyte system. Recombinant mAb 3B4 was expressed for 8 days by CHO-S cells growing in CD OptiCHO expression medium supplemented with 1 mM sodium butyrate, 8 mM L-glutamine, 1× HT supplement, and 0.1% Pluronic F68 at 32 °C with 125 rpm shaking. Every 24 h, cells were fed with CHO CD EfficientFeed A supplemented with 7 mM L-glutamine, 5.5% glucose, and 23.4 g/L yeastolate. After 8 days, cells were pelleted and medium containing secreted mAb 3B4 was diluted 1:1 with Pierce Protein A IgG Binding Buffer and 0.22-μM filtered. The sample was loaded onto a pre-equilibrated 1 mL HiTrap Protein A HP column. The column was washed with Pierce Protein A IgG Binding Buffer and elution was accomplished with low pH Pierce IgG Elution Buffer. Acidic elutions were neutralized by 2 M Tris pH 8.0 to 10% of the final volume.

### 2.6. Generation of Rabbit Polyclonal Serum to Human Astrovirus 1

Rabbit polyclonal serum to human astrovirus 1 was generated as described in [41].

### 2.7. Human Plasma Samples

Human plasma samples were obtained from Discovery Life Sciences. These 63 de-identified samples were collected from adults ages 19–78 (31 male, 32 female) between 2012–2016 (before the emergence of SARS-CoV-2). Plasma samples were heated at 56 °C for 1 h before use. Supplementary Table S1 includes information on patient age, sex, ethnicity, and race as well as date and location of sample collection.

### 2.8. Biolayer Interferometry Immunosorbent Assay

BLI-ISA studies were performed on an Octet RED384 instrument at 22 °C with shaking at 1000 rpm. BLI-ISA assay buffer consists of PBS pH 7.4, 2% BSA, and 0.1% Tween-20, which was 0.22-μm filtered. Before use, Anti-Penta-His (HIS1K) biosensors were placed into the wells of a biosensor-holding plate and pre-hydrated in BLI-ISA buffer for at least 10 min. Tilted-bottom 384-well microplates were loaded with 48 μL per well. The assay plate was prepared as follows: column 1 (BLI-ISA buffer), column 2 (10 nM His-tagged human astrovirus spikes 1–8 or 70 nM His-tagged SARS-CoV-2 RBD in BLI-ISA buffer), column 3 (a 1:4 dilution of ChonBlock in BLI-ISA buffer), column 4 (a 1:8 dilution of plasma samples: 6 μL plasma + 42 μL ChonBlock/BLI-ISA buffer), column 5 (BLI-ISA buffer), and column 6 (4 nm Colloidal Gold-AffiniPure Goat Anti-Human IgG or 4 nm Colloidal Gold-AffiniPure Goat Anti-Rabbit IgG secondary antibody diluted 1:10 in BLI-ISA buffer).

The BLI-ISA method was set as follows, with minimal adaptation from [37]. Baseline 1 (60 s) in column 1 (Equilibration), Loading (180 s) in column 2 (Antigen Loading: human astrovirus spikes 1–8 or SARS-CoV-2 RBD), Baseline 2 (60 s) in column 3 (Wash), Association 1 (600 s) in column 4 (Total Antibody Binding), Baseline 3 (60 s) in column 5 (Wash), and Association 2 (180 s) in column 6 (Detection: anti-human IgG-gold or anti-rabbit IgG-gold). Loading of 10 nM spikes or 70 nM SARS-CoV-2 RBD over 180 s onto Anti-Penta-His sensor tips resulted in a wavelength shift loading signal of ~0.55 nm.

Analysis of BLI-ISA data was performed and automated as previously described in [37].

## 3. Results

**Figure 2:**
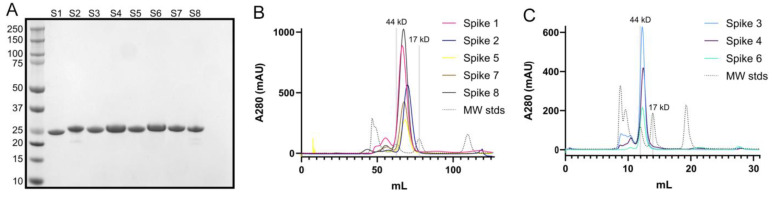
Purification of recombinant human astrovirus 1–8 capsid spikes (Spike 1–Spike 8). (**A**) Reducing SDS-PAGE of purified recombinant Spikes 1–8 (S1–S8). (**B**) Size exclusion chromatography column (Superdex 75 16/600) traces of Spikes 1, 2, 5, 7, and 8. (**C**) Size exclusion chromatography column (Superdex 75 10/300) traces of Spikes 3, 4, and 6. Size-exclusion chromatography confirmed that the purified spikes are folded and form dimers in solution.

First, we expressed recombinant Spike 1–Spike 8 antigens in *E. coli* and purified them by affinity chromatography and size exclusion chromatography. Purity was verified by SDS-PAGE (Figure 2A). Comparison of elution volumes for spike antigens to elution volumes for gel filtration standards confirmed that the spikes form dimers in solution (Figure 2B,C), consistent with structural studies [27,28,30].

**Figure 3:**
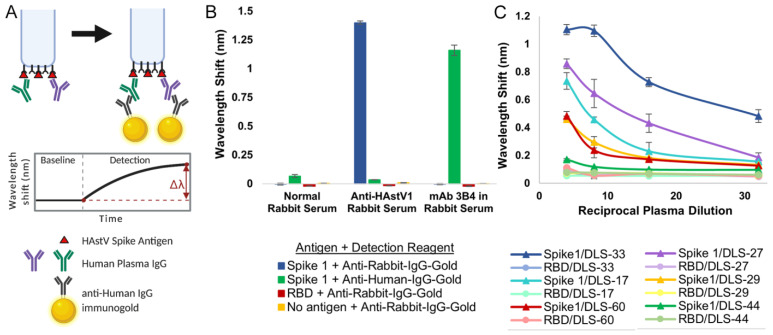
Validation of BLI-ISA for the detection of plasma IgG antibodies to human astrovirus capsid spikes. (**A**) BLI-ISA schematic of the IgG antibody detection step. This image was created with BioRender.com. (**B**) BLI-ISA detection of control sera. Spike 1, SARS-CoV-2 RBD, or no antigen were loaded onto biosensors and placed into 1:100 normal rabbit serum, 1:100 anti-human astrovirus 1 rabbit serum, or 25 nM mAb 3B4 in 1:100 normal rabbit serum. Bound antibodies were detected with anti-human-IgG-gold or anti-rabbit-IgG-gold. (**C**) Dilution-series BLI-ISA using Spike 1 (triangles) and RBD (circles) as antigens and representative strong astrovirus 1 seropositive (DLS-33, blue), moderate astrovirus 1 seropositive (DLS-17, cyan, and DLS-27, purple), weak astrovirus 1 seropositive (DLS-29, yellow, and DLS-60, red), and astrovirus 1 seronegative (DLS-44, green) plasma samples.

We then used the recombinant spike antigens in a biolayer interferometry immunosorbent assay (BLI-ISA). BLI-ISA is a recently developed method for rapid and semi-quantitative measurement of antibodies in blood plasma directed towards a particular antigen [37]. In a simple dip-and-read format, the binding of biomolecules to fiber-optic biosensors is measured in real time through a shift in the wavelength of the reflected light upon binding, with results available in 20 min. After an initial equilibration, the biosensors are loaded with the antigen of interest. Next, the antigen-loaded biosensors are washed and placed into diluted plasma to allow binding of antibodies to the antigen. After another wash, the antigen/antibody-coated biosensors are dipped into wells containing isotype-specific binding reagents, such as colloidal gold-conjugated anti-human IgG, and a wavelength shift detection signal is measured (Figure 3A). BLI-ISA was originally established as a tool for serosurveillance of SARS-CoV-2 [37]. During its development, known SARS-CoV-2 seropositive and seronegative plasma samples were first validated by a standard dilution-series enzyme-linked immunosorbent assay (ELISA) starting at a 1:50 dilution. Importantly, trends observed in subsequent 1:8 single-dilution BLI-ISA measurements of those samples correlated with area-under-the-curve calculations from the dilution-series ELISA, indicating that BLI-ISA can identify the variation in antibody levels in seropositive samples at this plasma dilution.

To confirm whether BLI-ISA can be applied to human astrovirus serosurveillance, we assessed Spike 1 antigen binding with commercially available normal rabbit serum and rabbit serum positive for human astrovirus serotype 1 [41]. As a positive control, monoclonal antibody (mAb) 3B4, a recombinant mouse–human chimeric antibody containing mouse variable regions that target Spike 1, was added to normal rabbit serum (Figure 3B). We also tested the specificity of our detection reagents, colloidal gold-conjugated anti-human IgG and colloidal gold-conjugated anti-rabbit IgG, in a 1:10 dilution. We loaded either Spike 1, the SARS-CoV-2 receptor-binding domain (RBD) negative control antigen, or no antigen onto the biosensor tips and dipped them into the indicated sera. Background signals were observed in all samples with a 1:100 dilution of normal rabbit serum, indicating a lack of anti-Spike 1 and anti-RBD antibodies in the serum, as well as low non-specific binding of the serum to the empty biosensor tip. When Spike 1 was loaded onto the biosensor and dipped into a 1:100 dilution of anti-human astrovirus 1 rabbit serum, a robust signal emerged when detected with the anti-rabbit-IgG-gold reagent (Figure 3B, blue) but not the anti-human-IgG-gold reagent, indicating the high specificity of the anti-rabbit-IgG-gold reagent. The background signal was observed for RBD-loaded and “No antigen” biosensors. Finally, we added 25 nM mAb 3B4 to a 1:100 dilution of normal rabbit serum into which we dipped Spike 1-loaded biosensors. We noted a strong signal when the sample was detected with the anti-human-IgG-gold reagent (Figure 3B, green) but not the anti-rabbit-IgG-gold reagent, showing the precision of the anti-human-IgG-gold reagent. Background signal was exhibited for RBD- and no antigen-loaded biosensors also in this case.

Next, we conducted dilution-series experiments to assess the optimal human plasma dilution to use in BLI-ISA serological assays for human astrovirus. Dzimianski et al. established a 1:8 plasma dilution as optimal after recent SARS-CoV-2 exposure, providing an estimate of the dilution range for serosurveillance studies using other antigens [37]. Sixty-three human plasma samples were collected between 2012–2016 (pre-SARS-CoV-2) from a randomized cross-sectional sample of the population in the United States, adults ages 19–78 (Supplementary Table S1). We performed dilution-series BLI-ISA on a representative subset of these plasma samples using Spike 1 as the antigen (Figure 3C). As a control for non-specific binding, the SARS-CoV-2 RBD was loaded onto the biosensors instead of Spike 1. The selected plasma samples include a strongly astrovirus 1-seropositive sample (DLS-33), two moderately astrovirus 1-seropositive samples (DLS-17 and DLS-27), two weakly astrovirus 1-seropositive samples (DLS-29 and DLS-60), and an astrovirus 1-seronegative sample (DLS-44) to represent the diversity of signals in a single-dilution assay. Each point was performed in duplicate. We loaded either Spike 1 or RBD onto the biosensors and then dipped into 1:4, 1:8, 1:16, or 1:32 dilutions of the indicated plasma. Detection signal at the 1:8 dilution compared to the 1:16 and 1:32 dilutions showed dose-dependent improvement for weak seropositives DLS-29 and DLS-60, as well as moderate seropositives DLS-17 and DLS-27 while maintaining low signal for the DLS-44 seronegative as well as the RBD-loaded background samples. While an even higher signal was observed for these weakly and moderately seropositive samples at the 1:4 dilution, compared to the 1:8 dilution, strong seropositive DLS-33 appears to reach signal saturation at the 1:8 dilution, with little signal improvement at the 1:4 dilution. In addition, seronegative sample DLS-44 and several of the RBD-loaded control samples showed non-specific signals at the 1:4 dilution but remained at background levels with the 1:8, 1:16, and 1:32 dilutions. Thus, we identified the 1:8 plasma dilution as optimal to maximize the dynamic range of the assay.

**Figure 4:**
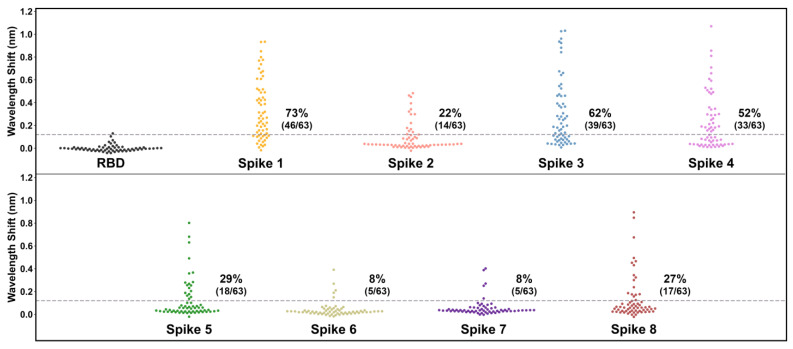
Human plasma IgG reactivity to human astrovirus spike proteins. Data are displayed as swarm plots, with each dot representing the signal for one individual for the indicated antigen. For samples with the RBD antigen, each sample’s RBD reactivity signal was subtracted by the mean RBD reactivity signal (0.07 nm) to center samples around zero. The dashed line indicates four standard deviations above zero for the RBD samples (0.12 nm), and the printed percentages denote the number of samples above the line. For samples with the spike antigens, each sample’s spike reactivity signal was subtracted by its own RBD reactivity signal to generate a background-corrected signal.

After validation of the control samples and confirmation of the optimal 1:8 plasma dilution, we used BLI-ISA to determine the seroprevalence of antibodies in the 63 human plasma samples to HAstV spikes from serotypes 1 to 8 (Figure 4 and Table 4). This investigation, using BLI-ISA to assess the unknown serostatus of individuals, is the first of its kind. Signals for each plasma sample to SARS-CoV-2 RBD were measured to establish each sample’s background detection value. To reduce inter-sample variability and prevent false positives from high-background samples, each sample’s spike reactivity signal was subtracted by its own RBD reactivity signal to generate a background-corrected detection value. The variability in plasma reactivity to RBD was used to estimate a seropositivity cut-off of four times the standard deviation of background binding to RBD. Overall, the percentage of samples containing IgG antibodies targeting the spike protein was the highest for human astrovirus serotype 1 (46/63, 73%), followed by serotype 3 (39/63, 62%), serotype 4 (33/63, 52%), serotype 5 (18/63, 29%), serotype 8 (17/63, 27%), serotype 2 (14/63, 22%), serotype 6 (5/63, 8%), and serotype 7 (5/63, 8%), which is in accordance with a comprehensive human astrovirus serosurveillance study conducted by Koopmans et al. in the Netherlands 23 years ago (Table 3) [10].

## 4. Discussion

Here, we evaluate the seroprevalence of plasma antibodies towards human astrovirus serotypes 1–8 in a cross-sectional sample of United States adults. We adapted BLI-ISA, a recently developed method for the rapid and semi-quantitative measurement of plasma antibodies, to measure IgG antibodies against recombinant human astrovirus 1–8 capsid spikes (Spike 1–Spike 8). We chose Spike 1–Spike 8 as antigens for their ease of production in *E. coli* and for their variable sequence identity (41.4–75.7%) which should minimize antibody cross-reactivity between spikes from different serotypes. Indeed, most known neutralizing monoclonal antibodies that target the spike are serotype specific [38,41,42], suggesting that total antibody levels against individual spikes should show minimal cross-reactivity. Plasma dilution series confirm that BLI-ISA is semi-quantitative, and a plasma dilution of 1:8 was selected as the ideal dilution to maximize the dynamic range of the BLI-ISA signals and minimize background signal using biosensors coated with a negative control antigen, the SARS-CoV-2 RBD, which should be non-reactive for antibodies in plasma collected before 2019 such as those in this study. Of note, a plasma dilution of 1:8 was also chosen to measure levels of antibodies to the SARS-CoV-2 RBD after recent SARS-CoV-2 exposure during BLI-ISA method development [37]. Time since exposure to human astrovirus is unknown for the samples in our study, yet the optimal plasma dilution remained 1:8. Therefore, BLI-ISA is a sensitive method that can detect antibodies elicited by an immune response which occurred potentially decades before plasma sample collection, while requiring very little plasma (6 μL total per sample per antigen).

This study estimates the seroprevalence rates, in order of prevalence, as 73% for HAstV1, 62% for HAstV3, 52% for HAstV4, 29% for HAstV5, 27% for HAstV8, 22% for HAstV2, 8% for HAstV6, and 8% for HAstV7. These findings are consistent with previous seroprevalence studies, as well as genotypic surveillance studies identifying human astrovirus 1 as the most prevalent serotype/genotype [9,10,11,12]. This result is also consistent with a previous seroprevalence study in identifying human astrovirus serotypes 3, 4, and 5 as having the next highest prevalence after serotype 1 (Table 3) [10]. We extend previous work by evaluating seroprevalence for human astrovirus serotype 8 for the first time and determine that it is relatively high at 27%, an unexpected observation given that it was discovered after serotypes 6 and 7, which have relatively low seroprevalence rates of 8% each. We note that seroprevalence rates in this study are likely modest estimates based upon a signal threshold of four times the standard deviation of the samples with a negative control antigen. Indeed, many individual samples had signals for spikes that were two or three times their own background signal yet did not meet the conservative criteria for seropositivity in this study.

Critically, seroprevalence rates for recombinant human astrovirus capsid spike antigens correlate closely with neutralizing antibody rates, determined previously (Table 3) [10]. This finding is significant because neutralizing antibodies are often a marker of protection from viral infection. While correlates of protection have yet to be defined for human astrovirus infection, there are some studies suggesting that antibodies are protective [23,24,25,26]. The observation that antibodies to the human astrovirus capsid spike correlate with virus neutralization is not entirely surprising, given previous evidence. Specifically, studies of HAstV-neutralizing monoclonal antibodies have identified that their epitopes reside on the spike domain or the capsid fragments that contains the spike domain, VP24 and VP26 [38,41,42,43]. Moreover, while both the capsid core and capsid spike domains stimulate antibody production in mice and rabbits, only antibodies to the spike show neutralizing activity [28,38,41]. Thus, plasma IgG levels against human astrovirus capsid spikes may provide a useful estimate of neutralizing antibody levels.

Finally, we present Table 4 as a heatmap of antibody levels of each individual for human astrovirus 1–8 capsid spikes. Individuals were clustered using the Ward distance minimization algorithm to identify trends in seropositivity. We find that every individual has reactivity to at least one serotype of spike and, on average, individuals are seropositive for about 3 human astrovirus serotypes. While no sample had reactivity to all 8 spikes, one sample had reactivity to 7 spikes (DLS-15). A few samples were only weakly to moderately seropositive to only one or two spikes (DLS-13, -57, -60). We examined the data for correlations in sample positivity between any two serotypes, indicating potential antibody cross-reactivity, but did not identify any trends. Interestingly, clustering revealed a trend in individuals with high seroreactivity for one spike from either HAstV1, -3, or -4 (which have the highest seroprevalence) in that these individuals rarely have high seroreactivity to another spike from these prevalent serotypes. This result might indicate that a strong antibody response to a first infection provides partial protection to an infection by a different serotype, resulting in a weaker antibody response to the different serotype. However, further research is necessary to explore this possibility.

## Figures and Tables

**Table 1:** Sequence identity matrix across capsid core domains from human astrovirus serotypes 1–8.

	Core 1	Core 2	Core 3	Core 4	Core 5	Core 6	Core 7	Core 8
Core 1	100%	86.7%	91.5%	86.1%	90.0%	90.9%	87.9%	86.7%
Core 2	-	100%	86.7%	87.9%	83.3%	83.9%	85.2%	87.9%
Core 3	-	-	100%	87.3%	90.6%	91.2%	92.8%	88.2%
Core 4	-	-	-	100%	84.6%	86.1%	84.9%	97.0%
Core 5	-	-	-	-	100%	90.3%	88.8%	85.8%
Core 6	-	-	-	-	-	100%	87.9%	87.6%
Core 7	-	-	-	-	-	-	100%	85.8%
Core 8	-	-	-	-	-	-	-	100%

**Table 2:** Sequence identity matrix across capsid spike domains from human astrovirus serotypes 1–8.

	Spike 1	Spike 2	Spike 3	Spike 4	Spike 5	Spike 6	Spike 7	Spike 8
Spike 1	100%	50.2%	61.2%	41.4%	47.0%	53.1%	58.9%	53.0%
Spike 2	-	100%	46.7%	47.9%	41.8%	47.0%	44.9%	49.8%
Spike 3	-	-	100%	46.3%	48.6%	54.7%	75.7%	55.1%
Spike 4	-	-	-	100%	46.0%	47.0%	43.5%	46.1%
Spike 5	-	-	-	-	100%	63.34%	52.8%	52.1%
Spike 6	-	-	-	-	-	100%	56.6%	57.3%
Spike 7	-	-	-	-	-	-	100%	53.3%
Spike 8	-	-	-	-	-	-	-	100%

**Table 3:** Summary of previous human astrovirus serological surveys.

Study Year Published	Seroprevalence	Size	Antigen
Koopmans et al. [10] 1998	Ages 0–79: Human astrovirus 1: 91% Human astrovirus 2: 31% Human astrovirus 3: 69% Human astrovirus 4: 56% Human astrovirus 5: 36% Human astrovirus 6: 16% Human astrovirus 7: 10% Human astrovirus 8: ---	242 people ages 0–79	Human astrovirus particles cultured in Caco-2 cells
Mitchell et al. [31] 1999	By age 9: Human astrovirus 1: 94% Human astrovirus 3: 42%	393 children	Baculovirus-expressed recombinant capsid proteins
Kriston et al. [32] 1996	By age 5: Human astrovirus 1: 90% Human astrovirus 6: 10–30%	273 children and hospital staff	Baculovirus-expressed recombinant capsid proteins
Kobayashi et al. [33] 1999	By age 3: Human astrovirus 1: ~100% Human astrovirus 2: ~100%	170 children	Unclear: Article in Japanese
Kurtz et al. [23] 1978	Presence of “astrovirus antibody” By 6 months: 45% By age 1: 7% By age 2: 25% By age 4: 71% By age 10: 75% Young adult cohort: 77%	87 children ages 0–10 70 young adults ages 17–30	Human astrovirus particles cultured in HEK cells
Holtz et al. [34] 2014	By age 17: MLB1: 100%	395 people ages 0–95	Baculovirus-expressed recombinant capsid protein
Burbelo et al. [35] 2011	HMOAstV-C (VA1): 36% by age 10 65% in adults	103 children and 106 adults	Crude protein extracts containing N- and C-terminal capsid fragments
Meliopoulos et al. [36] 2014	By adulthood: Human astrovirus 1: 81% Turkey astrovirus 2: 26%	160 turkey growers, turkey meat processing plant workers, and unexposed workers	Baculovirus-expressed recombinant capsid proteins

**Table 4:** Heat map of individual human plasma IgG reactivity to human astrovirus spike antigens. ^1^

		Controls		Human Astrovirus Spike Antigens (Background-Corrected)							
	ID	No Antigen	RBD	Spike 1	Spike 2	Spike 3	Spike 4	Spike 5	Spike 6	Spike 7	Spike 8
1	DLS-18	0.13	0.06	0.11	0.04	1.03	0.03	0.02	0.00	0.05	0.03
2	DLS-14	0.10	0.05	0.20	0.03	0.88	0.15	0.03	0.03	0.09	0.07
3	DLS-55	0.07	0.06	0.32	0.15	0.92	0.04	0.04	0.06	0.10	0.18
4	DLS-44	0.05	0.04	0.02	0.03	0.46	0.15	0.02	0.05	0.04	0.17
5	DLS-36	0.14	0.07	0.01	0.09	0.54	0.03	0.01	0.01	0.03	0.06
6	DLS-51	0.08	0.07	0.16	0.02	0.66	0.01	0.02	0.01	0.04	0.02
7	DLS-06	0.11	0.06	0.11	0.04	0.67	0.53	0.06	0.03	0.05	0.09
8	DLS-01	0.14	0.08	0.03	0.11	0.96	0.59	0.03	0.04	0.06	0.32
9	DLS-30	0.13	0.09	0.43	0.02	0.31	0.09	0.26	0.03	0.07	0.07
10	DLS-02	0.20	0.10	0.52	0.03	0.47	0.09	0.27	0.05	0.05	0.10
11	DLS-41	0.10	0.07	0.68	0.03	0.52	0.22	0.28	0.19	0.08	0.08
12	DLS-20	0.09	0.05	0.44	0.03	0.46	0.03	0.03	0.02	0.04	0.03
13	DLS-05	0.37	0.20	0.42	−0.02	0.56	0.07	0.16	0.00	0.01	−0.02
14	DLS-54	0.07	0.07	0.31	0.01	0.37	0.03	0.03	0.03	0.09	0.02
15	DLS-21	0.22	0.17	0.27	0.02	0.46	0.03	−0.02	0.00	0.04	0.12
16	DLS-49	0.09	0.06	0.78	0.02	0.84	0.02	0.06	0.02	0.25	0.06
17	DLS-35	0.09	0.07	0.63	0.14	0.64	0.19	0.01	0.01	0.02	0.01
18	DLS-24	0.12	0.06	0.11	0.03	1.03	0.07	0.19	0.06	0.27	0.89
19	DLS-19	0.08	0.08	0.12	0.00	0.18	0.01	0.14	0.00	0.39	0.47
20	DLS-28	0.06	0.03	0.19	0.03	0.28	0.04	0.02	0.01	0.02	0.67
21	DLS-42	0.07	0.06	0.26	0.03	0.13	0.01	0.02	0.01	0.04	0.43
22	DLS-07	0.09	0.05	0.26	0.01	0.08	0.10	0.68	0.05	0.03	0.05
23	DLS-25	0.10	0.06	0.85	0.46	0.05	0.12	0.63	0.03	0.03	0.09
24	DLS-27	0.11	0.08	0.77	0.01	0.06	0.08	0.02	−0.01	0.01	0.34
25	DLS-34	0.10	0.07	0.70	0.01	0.14	0.03	0.03	0.00	0.02	0.45
26	DLS-23	0.09	0.05	0.93	0.09	0.28	0.07	0.36	0.03	0.04	0.04
27	DLS-33	0.10	0.10	0.93	0.03	0.08	0.01	0.03	−0.02	0.00	0.00
28	DLS-09	0.29	0.12	0.80	0.07	0.10	0.17	0.05	0.07	0.02	0.02
29	DLS-53	0.08	0.06	0.74	0.03	0.04	0.29	0.10	0.06	0.04	0.08
30	DLS-61	0.08	0.07	0.49	0.01	0.03	0.02	0.06	0.01	0.03	0.01
31	DLS-17	0.09	0.06	0.61	0.03	0.19	0.03	0.02	0.01	0.04	0.03
32	DLS-04	0.09	0.04	0.49	0.03	0.25	0.05	0.07	0.02	0.06	0.06
33	DLS-46	0.08	0.06	0.39	0.02	0.17	0.03	0.03	0.02	0.03	0.02
34	DLS-12	0.06	0.03	0.38	0.03	0.11	0.03	0.25	0.03	0.03	0.05
35	DLS-31	0.16	0.14	0.40	0.01	0.07	0.18	0.08	−0.01	0.01	0.00
36	DLS-40	0.09	0.05	0.31	0.02	0.06	0.21	0.07	0.03	0.03	0.03
37	DLS-26	0.12	0.08	0.61	0.04	0.20	0.26	0.06	0.00	0.02	0.17
38	DLS-45	0.10	0.07	0.51	0.12	0.28	0.25	0.07	0.02	0.06	0.30
39	DLS-39	0.13	0.07	0.45	0.17	0.10	0.36	0.02	0.02	0.06	0.06
40	DLS-47	0.09	0.05	0.66	0.01	0.04	0.52	0.03	0.02	0.03	0.02
41	DLS-59	0.08	0.07	0.21	0.02	0.09	0.49	0.03	0.03	0.40	0.02
42	DLS-32	0.11	0.07	0.07	0.00	0.13	0.49	0.01	0.02	0.01	0.07
43	DLS-37	0.11	0.07	0.09	0.03	0.36	0.35	0.05	0.02	0.04	0.24
44	DLS-43	0.10	0.06	0.09	0.03	0.36	0.30	0.03	0.02	0.03	0.04
45	DLS-15	0.08	0.04	0.22	0.08	0.25	0.34	0.23	0.21	0.14	0.16
46	DLS-58	0.08	0.06	0.28	0.22	0.39	0.48	0.04	0.03	0.04	0.12
47	DLS-11	0.08	0.04	0.07	0.45	0.03	0.19	0.18	0.02	0.03	0.06
48	DLS-62	0.13	0.12	0.10	0.32	0.26	0.07	0.03	0.05	0.08	0.08
49	DLS-57	0.07	0.06	0.04	0.04	0.04	0.16	0.04	0.02	0.03	0.19
50	DLS-13	0.09	0.04	0.03	0.12	0.19	0.07	0.15	0.01	0.04	0.05
51	DLS-48	0.09	0.06	0.06	0.09	0.22	0.18	0.07	0.02	0.03	0.02
52	DLS-56	0.07	0.08	0.14	0.34	0.93	0.71	0.80	0.27	0.01	0.11
53	DLS-63	0.07	0.08	0.11	0.48	0.39	0.51	0.49	0.15	0.03	0.04
54	DLS-08	0.08	0.05	0.52	0.30	0.11	1.07	0.28	0.39	0.03	0.85
55	DLS-52	0.22	0.11	−0.02	0.39	0.01	0.81	0.01	0.00	0.00	0.49
56	DLS-50	0.10	0.07	0.42	0.18	0.12	0.66	0.37	0.05	0.04	0.09
57	DLS-03	0.08	0.04	0.17	0.04	0.04	0.85	0.20	0.07	0.03	0.05
58	DLS-22	0.11	0.06	0.16	0.30	0.07	0.61	0.08	0.03	0.06	0.04
59	DLS-29	0.08	0.05	0.20	0.04	0.04	0.18	0.06	0.02	0.03	0.02
60	DLS-10	0.10	0.05	0.22	0.10	0.05	0.30	0.10	0.04	0.03	0.06
61	DLS-16	0.09	0.04	0.13	0.08	0.08	0.29	0.03	0.02	0.03	0.03
62	DLS-38	0.16	0.08	0.24	0.01	0.15	0.03	0.01	0.01	0.02	0.02
63	DLS-60	0.05	0.04	0.13	0.01	0.03	0.01	0.04	0.03	0.02	0.02
64	PBS	0.06	0.05	0.01	0.02	0.02	0.02	0.02	0.03	0.02	0.02

^1^ For samples with no antigen and the RBD antigen, the true reactivity signals are reported. For the spike samples, each sample’s spike reactivity signal was subtracted by its own RBD reactivity signal to generate a background-corrected signal. To aid the visualization of patterns in human astrovirus reactivity, spike reactivity values were hierarchically clustered using a SciPy python library function, wherein distance was calculated with the Ward variance minimization algorithm (scipy.cluster.hierarchy.linkage(spike-data, method = ‘ward’)). After initial clustering, minor additional sorting was performed and reactivity values were colored according to their magnitude to produce the heatmap.

## Data Availability

The data presented in this study are available within the article and its Supplementary Material at https://www.mdpi.com/article/10.3390/v13060979/s1.

## Supplementary Materials

The following are available online at https://www.mdpi.com/article/10.3390/v13060979/s1, Table S1: Plasma Samples from Discovery Life Sciences.
