# Supplementary material for: Human Astrovirus 1–8 Seroprevalence Evaluation in a United States Adult Population
Source: Viruses. 2021 May 25;13(6):979. doi: 10.3390/v13060979 (PMC8229645; doi:10.3390/v13060979)
Supplement: Supplementary file 1 [file viruses-13-00979-s001.zip › viruses-1206515-supplementary.pdf]

SUPPLEMENTARY TABLE 1. Plasma Samples from Discovery Life Sciences

Internal ID	*Patient ID*	*Sample SKU*	*Aliquot Serial*	*Gender*	*Age*	*Race*	*Ethnicity*	*Region*	*Sample Draw Date*
DLS-1	110033775	BBP0500-A5110033775072514P0	173754487	Male	49	Black	Non-Hispanic/Latino	Huntsville, AL, USA	7/25/14
DLS-2	110032786	BBP0500-A5110032786072313P0	150952714	Male	59	White	Non-Hispanic/Latino	Huntsville, AL, USA	7/23/13
DLS-3	110000224	BBP0500-A5110000224072313P0	156150353	Male	66	White	Non-Hispanic/Latino	Huntsville, AL, USA	7/23/13
DLS-4	110027059	BBP0500-A5110027059110813P0	156151663	Female	35	White	Non-Hispanic/Latino	Huntsville, AL, USA	11/8/13
DLS-5	110000219	BBP0500-A5110000219110713P0	156151819	Male	65	Black	Non-Hispanic/Latino	Huntsville, AL, USA	11/7/13
DLS-6	110035404	BBP0500-A5110035404080613P0	150954519	Male	70	White	Non-Hispanic/Latino	Huntsville, AL, USA	8/6/13
DLS-7	110033582	BBP0500-A5110033582012714P0	159302610	Male	38	White	Non-Hispanic/Latino	Huntsville, AL, USA	1/27/14
DLS-8	110000291	BBP0500-A5110000291012214P0	158368339	Female	56	White	Hispanic/Latino	Huntsville, AL, USA	1/22/14
DLS-9	110027066	BBP0500-A5110027066110513P0	145973823	Male	32	White	Non-Hispanic/Latino	Huntsville, AL, USA	11/5/13
DLS-10	110033642	BBP0500-A5110033642020514P0	159302017	Female	50	White	Non-Hispanic/Latino	Huntsville, AL, USA	2/5/14
DLS-11	110033707	BBP0500-A5110033707021414P0	162046387	Female	36	White	Hispanic/Latino	Huntsville, AL, USA	2/14/14
DLS-12	110033646	BBP0500-A5110033646021414P0	158386097	Male	39	White	Non-Hispanic/Latino	Huntsville, AL, USA	2/14/14
DLS-13	110027070	BBP0500-A5110027070080812P0	128358537	Male	40	Black	Non-Hispanic/Latino	Huntsville, AL, USA	8/8/12
DLS-14	110035403	BBP0500-A5110035403081613P0	156827302	Male	65	White	Hispanic/Latino	Huntsville, AL, USA	8/16/13
DLS-15	110033711	BBP0500-A5110033711030514P0	162047215	Female	45	White	Non-Hispanic/Latino	Huntsville, AL, USA	3/5/14
DLS-16	110035415	BBP0500-A5110035415081313P0	152418158	Male	68	White	Non-Hispanic/Latino	Huntsville, AL, USA	8/13/13
DLS-17	110032789	BBP0500-A5110032789111513P0	156152366	Female	23	White	Non-Hispanic/Latino	Huntsville, AL, USA	1/31/14
DLS-18	110035416	BBP0500-A5110035416100713SH	141531749	Male	72	White	Non-Hispanic/Latino	Huntsville, AL, USA	10/7/13
DLS-19	110029613	BBP0500-A5110029613112113P0	141502563	Male	56	Black	Non-Hispanic/Latino	Huntsville, AL, USA	11/21/13
DLS-20	110033565	BBP0500-A5110033565112213P0	141504119	Male	30	Asian	Non-Hispanic/Latino	Huntsville, AL, USA	11/22/13
DLS-21	110033572	BBP0500-A5110033572112213P0	141504164	Female	22	White	Hispanic/Latino	Huntsville, AL, USA	11/22/13
DLS-22	110033569	BBP0500-A5110033569112213P0	141504138	Male	35	White	Hispanic/Latino	Huntsville, AL, USA	11/22/13
DLS-23	110000296	BBP0500-A5110000296071913P0	149460809	Male	53	White	Non-Hispanic/Latino	Huntsville, AL, USA	7/19/13
DLS-24	110029648	BBP0500-A5110029648110613P0	145974097	Male	66	Black	Non-Hispanic/Latino	Huntsville, AL, USA	11/6/13
DLS-25	110033618	BBP0500-A5110033618022014P0	162046318	Female	28	Other	Non-Hispanic/Latino	Huntsville, AL, USA	2/20/14
DLS-26	110033587	BBP0500-A5110033587121313P0	158367340	Female	28	Black	Non-Hispanic/Latino	Huntsville, AL, USA	12/13/13
DLS-27	110033761	BBP0500-A5110033761061914P0	174556350	Male	25	Black	Non-Hispanic/Latino	Huntsville, AL, USA	6/19/14
DLS-28	110033765	BBP0500-A5110033765062314P0	174555855	Female	34	White	Non-Hispanic/Latino	Huntsville, AL, USA	6/23/14
DLS-29	110033735	BBP0500-A5110033735041414P0	162034611	Female	41	Black	Non-Hispanic/Latino	Huntsville, AL, USA	4/14/14
DLS-30	110033918	BBP0500-A5110033918092214P0	176336325	Female	39	Asian	Non-Hispanic/Latino	Huntsville, AL, USA	9/22/14
DLS-31	110029632	BBP0500-A5110029632071713P0	149460542	Male	78	White	Non-Hispanic/Latino	Huntsville, AL, USA	7/17/13
DLS-32	110027067	BBP0500-A5110027067101716P0	219566034	Male	50	White	Non-Hispanic/Latino	Huntsville, AL, USA	10/17/16
DLS-33	110036064	BBP0500-A5110036064112315P0	202006509	Male	20	Black	Non-Hispanic/Latino	Huntsville, AL, USA	11/23/15
DLS-34	110036542	BBP0500-Z1110036542020916SH	202065896	Female	22	White	Non-Hispanic/Latino	Huntsville, AL, USA	2/9/16
DLS-35	110000239	BBP0500-A5110000239111113P0	145973691	Female	63	White	Non-Hispanic/Latino	Huntsville, AL, USA	11/11/13
DLS-36	110033246	BBP0500-A5110033246072513P0	150953001	Male	52	White	Hispanic/Latino	Huntsville, AL, USA	7/25/13

DLS-37	110000267	BBP0500-A5110000267080912P0	128359003	Male	63	White	Unknown	Huntsville, AL, USA	8/9/12
DLS-38	110033586	BBP0500-A5110033586121113P0	158366754	Female	30	White	Non-Hispanic/Latino	Huntsville, AL, USA	12/11/13
DLS-39	110033594	BBP0500-A5110033594121113P0	158366732	Female	22	White	Non-Hispanic/Latino	Huntsville, AL, USA	12/11/13
DLS-40	110029622	BBP0500-A5110029622011314P0	159304314	Female	56	White	Non-Hispanic/Latino	Huntsville, AL, USA	1/13/14
DLS-41	110033624	BBP0500-A5110033624011714P0	159301735	Male	55	White	Non-Hispanic/Latino	Huntsville, AL, USA	1/17/14
DLS-42	110033395	BBP0500-A5110033395111313P0	145973555	Female	29	Black	Non-Hispanic/Latino	Huntsville, AL, USA	11/13/13
DLS-43	110033584	BBP0500-A5110033584120613P0	158366873	Female	28	Black	Non-Hispanic/Latino	Huntsville, AL, USA	12/6/13
DLS-44	110033643	BBP0500-A5110033643020414P0	159302402	Male	22	White	Hispanic/Latino	Huntsville, AL, USA	2/4/14
DLS-45	110033590	BBP0500-A5110033590010814P0	158386007	Female	25	White	Non-Hispanic/Latino	Huntsville, AL, USA	1/8/14
DLS-46	110000293	BBP0500-A5110000293011014P0	159303362	Male	29	White	Non-Hispanic/Latino	Huntsville, AL, USA	1/10/14
DLS-47	110033585	BBP0500-A5110033585010314P0	158369210	Male	20	White	Non-Hispanic/Latino	Huntsville, AL, USA	1/3/14
DLS-48	110033622	BBP0500-A5110033622010214P0	158366769	Male	29	Black	Non-Hispanic/Latino	Huntsville, AL, USA	1/2/14
DLS-49	110033640	BBP0500-A5110033640012914P0	159303038	Male	43	Asian	Non-Hispanic/Latino	Huntsville, AL, USA	1/29/14
DLS-50	110033626	BBP0500-A5110033626123013P0	158385555	Male	19	White	Non-Hispanic/Latino	Huntsville, AL, USA	12/30/13
DLS-51	110033628	BBP0500-A5110033628123113P0	158369324	Female	25	White	Non-Hispanic/Latino	Huntsville, AL, USA	12/31/13
DLS-52	110033570	BBP0500-A5110033570010314P0	158369292	Female	26	White	Non-Hispanic/Latino	Huntsville, AL, USA	1/3/14
DLS-53	110033632	BBP0500-A5110033632011514P0	159301330	Female	30	American Indian	Non-Hispanic/Latino	Huntsville, AL, USA	1/15/14
DLS-54	110033235	BBP0500-A5110033235121913P0	158385538	Female	21	White	Non-Hispanic/Latino	Huntsville, AL, USA	12/19/13
DLS-55	110033621	BBP0500-A5110033621122713P0	158386170	Female	26	American Indian	Non-Hispanic/Latino	Huntsville, AL, USA	12/27/13
DLS-56	110033770	BBP0500-A5110033770062514P0	174555254	Female	33	White	Non-Hispanic/Latino	Huntsville, AL, USA	6/25/14
DLS-57	110033747	BBP0500-A5110033747050514P0	158382857	Female	48	Black	Non-Hispanic/Latino	Huntsville, AL, USA	5/5/14
DLS-58	110033767	BBP0500-A5110033767070714P0	174555619	Female	44	Black	Non-Hispanic/Latino	Huntsville, AL, USA	7/7/14
DLS-59	110033243	BBP0500-A5110033243052214P0	162047964	Female	38	Black	Non-Hispanic/Latino	Huntsville, AL, USA	5/22/14
DLS-60	110033759	BBP0500-A5110033759061714P0	174556297	Female	40	Black	Non-Hispanic/Latino	Huntsville, AL, USA	6/17/14
DLS-61	110033644	BBP0500-A5110033644061214P0	173753188	Female	32	Black	Non-Hispanic/Latino	Huntsville, AL, USA	6/12/14
DLS-62	110033721	BBP0500-A5110033721043014P0	162047783	Female	27	Black	Non-Hispanic/Latino	Huntsville, AL, USA	4/30/14
DLS-63	110033915	BBP0500-A5110033915082814P0	176337760	Male	43	Black	Non-Hispanic/Latino	Huntsville, AL, USA	8/28/14
